# Effectiveness of Surgical Periodontal Therapy in Oral Health-Related Quality of Life

**DOI:** 10.7759/cureus.58792

**Published:** 2024-04-22

**Authors:** Swarna Meenakshi P, Arvina Rajasekar

**Affiliations:** 1 Periodontics, Saveetha Dental College and Hospitals, Saveetha Institute of Medical and Technical Sciences, Saveetha University, Chennai, IND

**Keywords:** oral health, flap surgery, surgical periodontal therapy, surrogate end points, true end points

## Abstract

Background and objective

The objective of this study is to evaluate and compare the surrogate and true end points following surgical periodontal therapy using the Oral Health Impact Profile-14 (OHIP-14) questionnaire.

Materials and methods

The study included a total of 30 participants, comprising 15 males and 15 females aged between 25 and 50 years. All individuals who had undergone periodontal flap surgery for generalized chronic periodontitis at the Department of Periodontology, Saveetha Dental College and Hospitals were included in the study. The OHIP-14 questionnaire was used to assess the patient-centered outcomes (true end points) pre- and post-flap surgery at baseline and six months. Surrogate end points such as the clinical attachment level (CAL), probing pocket depth (PPD), and gingival index (GI) were recorded at baseline and six months pre- and post-flap surgery.

Results

Clinical parameters such as the GI (p=0.03*), CAL (p=0.03), and PPD (p=0.02*) showed a statistically significant improvement after surgery. Patient-centered outcomes showed statistically significant differences in terms of taste perception, reduction in pain sensation, improvement in self-consciousness and reduction in anxiety levels, diminution of the feeling of embarrassment and enhancement in the ability to relax due to problems associated with gums, and improvement in the workplace (p<0.05) post-operatively.

Conclusion

Surgical periodontal therapy plays a pivotal role in improving oral health-related quality of life (OHRQoL) among patients with chronic periodontal disease. Utilizing OHIP-14 as an assessment tool enables a comprehensive evaluation of treatment outcomes, encompassing various dimensions of oral health impact. Patient-centered outcomes such as psychological discomfort and functional limitations can be achieved only by an interdisciplinary approach.

## Introduction

Periodontitis is classically recognized by the gradual deterioration of both the soft and hard tissues of the periodontal complex [1]. The progression of this disease involves a dynamic interplay between the microbial communities, characterized by dysbiosis, and the host's immune reactions occurring within the gingival and periodontal tissues. The intricate nature of this interaction contributes to the breakdown of periodontium which comprises gingiva, cementum, alveolar bone, and periodontal ligament resulting in bleeding gums, halitosis, gingival recession, and thereby leading to tooth loss if left untreated [2-3].

As per the findings of the Global Burden of Disease Study in 2016, severe periodontal disease ranked as the 11th most widespread health condition globally [4]. The prevalence of periodontal disease was documented to vary between 20% and 50% around the world [5]. Notably, it stands out as a significant contributor to tooth loss, posing potential impacts on essential functions such as mastication, aesthetic concerns, self-confidence, and overall quality of life [6]. Timely identification coupled with appropriate intervention plays a crucial role in the effective management of periodontal conditions, aiding in the reduction of its impact on oral health. Fundamental to the prevention and control of periodontal disease are routine dental examinations, professional cleanings, and early intervention which play an integral role in controlling the progression of the disease. The primary modalities for treating periodontitis encompass non-surgical strategies and surgical interventions [7]. Surgical interventions are indicated in those patients who have residual pockets that may jeopardize the survival of the tooth and be a risk factor for further progression of the disease [8].

Evidence-based dentistry encompasses four distinct parameters: patient values, scientific evidence, clinical knowledge and experience, and judgment. These parameters collectively contribute to informed decision-making, and treatment planning, and serve as a foundation for future research endeavors [9]. Among these elements, patient values hold particular significance as the patient is the primary beneficiary of the treatment. Acknowledging and prioritizing the patient's perspective on changes resulting from the treatment process is crucial. Therefore, any treatment or intervention can be regarded as lucrative if it meets true end points. This strategy is vital for attaining treatment goals that extend beyond purely clinical metrics and are in harmony with the overall well-being and quality of life of the patient [10].

True clinical end points in periodontology should gauge a patient's emotions, functionality, or survival. These end points mainly highlight the patient’s comprehension of the treatment. True end points gauge a patient's tangible subjective response, directly correlating with the patient's quality of life [11]. The proposition was made that assessments of subjective oral health-related quality of life should be regarded as true end points for evaluating the effectiveness of periodontal treatments. On the other hand, surrogate end points such as the gingival index (GI), probing pocket depth (PPD), and clinical attachment level (CAL) are objective outcomes of disease activity and the efficacy of the treatment but may not directly influence the patient’s experience [12]. The differentiation between true clinical end points and surrogate end points holds significance as enhancements in surrogate measurements signify positive shifts in disease activity. However, it remains unclear whether these improvements consistently translate into an enhanced quality of life for the patient. Therefore, it is yet to be substantiated with evidence whether enhancements in surrogate measurements correspond to an enhanced quality of life concerning dental comfort, functionality, appearance, and social performance.

Clinically, the assessment of periodontal flap surgery should encompass the reduction in various parameters including halitosis, sensitivity, bleeding on probing, tooth mobility, and food impaction. It should also gauge the improvement in the functioning of teeth and evaluate enhancements related to the patient's primary complaint as outcome parameters. This approach is preferred over solely relying on surrogate endpoints. 

In the realm of periodontics, only a limited number of trials have incorporated true end points as outcome measures. Notably, two trials have provided evidence supporting the use of clinical attachment loss as a reliable surrogate for predicting tooth loss [13-14]. However, an extensive literature search has yielded no trials within periodontics that validate the utilization of surrogate end points. Health extends beyond the mere absence of disease, and the objectives of healthcare encompass not only the mitigation of clinical disease indicators but also the alleviation of symptoms, disability, and enhancement of the quality of life. These aspects, which can only be evaluated by the patients themselves, underscore the necessity for patient-centered outcomes originally referred to as socio-dental indicators, the importance of incorporating such outcomes has been widely endorsed in both research and clinical settings. Therefore, assessing the influence of periodontal flap surgery on outcomes that are centered around the patient is a crucial aspect of evaluating an individual's health requirements. 

 The Oral Health Impact Profile (OHIP) is a questionnaire that measures people’s perception of the social impact of oral disorders on their well-being [15]. Slade in 1997 developed a modified form of it with 14 questions, named OHIP-14 which showed good reliability, validity, and precision [16]. Fourteen items of OHIP are divided into seven dimensions: functional limitation, physical discomfort, psychological discomfort, physical disability, psychological disability, social disability, and handicaps. Therefore, the study aims to assess the clinical and patient-centered outcomes to evaluate the influence of surgical periodontal therapy on oral health.

## Materials and methods

Study population

This study included a total of 30 participants, comprising 15 males and 15 females aged between 25 and 50 years. All individuals who had undergone periodontal flap surgery for generalized chronic periodontitis at the Department of Periodontology, Saveetha Dental College and Hospitals, Chennai were included in this study. All the patients included in the study were explained about the questionnaire and signed an informed consent before participating in the study. The study design and protocol were duly approved and reviewed by the Institutional Ethical Committee (IHEC/SDC/PERIO-2102/24/015).

Inclusion criteria

The participants included in the study met the following criteria: systemically healthy patients; patients who do not have any habits like smoking and tobacco chewing; periodontitis patients with PPD values exceeding 5mm in more than 30% of sites; CAL values exceeding 5mm in more than 30% sites (moderate to severe chronic periodontitis); full mouth radiographic periapical examination showing periodontal bone loss; and patients with no history of drug intake that affects periodontium.

Exclusion criteria

Participants with poor oral hygiene measures, pregnant women, immunologically compromised patients, and medically compromised patients were excluded from the study.

Surgical periodontal therapy

The treatment protocol involved surgical periodontal therapy to gain access to osseous defects and to eliminate infrabony pockets and interdental craters. Sulcular and crestal incisions were given by Bard-Parker blade 15 followed by which the flap was elevated using a P-24G elevator and the underlying bone was exposed. Granulation tissue was removed using area-specific curettes with specific numbers (1/2, 3/4, 11/12, 13/14, HU-friedy). Suturing was done with non-resorbable silk sutures. Post-operative instructions were given after flap surgery.

Outcomes measured

Clinical Parameters

Periodontal parameters (GI, PPD, CAL) were recorded at baseline and six months post-surgical periodontal therapy using the WHO periodontal probe. The GI was recorded using the Loe and Silness Index, the PPD was recorded from the free gingival margin to the base of the sulcus and the CAL was recorded from the cementoenamel junction to the base of the sulcus.

Patient-Centered Outcomes

Patient-centered outcomes were recorded at baseline and six months post-surgical periodontal therapy using the OHIP-14 questionnaire. Each question was framed as an opinion statement, utilizing the Likert scale and employing a 5-point response format (strongly disagree, agree, neutral, strongly agree, and agree). Responses were rated on a scale from always (scored 5) to never (scored 0).

Statistical analysis

The data collected were entered into a Microsoft Excel spreadsheet and analyzed using IBM SPSS Statistics for Windows, Version 23 (Released 2015; IBM Corp., Armonk, New York, United States). Descriptive statistics, including mean and standard deviation, were utilized for presenting data related to the gingival index, pocket probing depth, and clinical attachment level. A paired t-test was done to compare these parameters between baseline and at six months. Frequency distribution and percentage were employed to analyze the questionnaire. To test the statistical significance of responses before and after surgery, the Wilcoxon paired signed-rank test was applied.

## Results

The results of the current study after six months of surgical periodontal therapy, incorporating the GI, PPD, and CAL, are presented in Table 1. All the parameters showed a significant improvement between pre- and post-operative flap surgery (p<0.05) (Table 1).

**Table 1:** Comparison of clinical parameters pre- and post-surgical periodontal therapy. A statistically significant difference was observed in all the clinical parameters. The gingival index showed a statistically significant difference of p=0.03*. The probing pocket depth (PPD) showed a statistically significant difference of p=0.02*. Clinical attachment loss (CAL) showed a statistically significant difference of p=0.03*.

	Gingival Index		PPD		CAL	
	Pre	Post	Pre	Post	Pre	Post
Mean	2.1867	1.1933	8.0987	5.9370	8.3260	6.0003
Standard Deviation	0.42809	0.36097	0.80045	0.46312	0.728530	0.52678
p-value	0.03*		0.02*		0.03*	

The results of the patient-centered responses were evaluated under seven domains with 14 questions. In the functional domain regarding pronunciation, 100% of patients had problems with pronunciation prior to the flap surgery whereas only 20% of the patients were satisfied even after flap surgery. Regarding the altered taste perception before surgery, 100% of the patients had poor taste perception and 80% of the patients had significant enhancement in taste perception post-operatively. The physical pain domain assessed painful sensation and discomfort in eating food because of problems with gums. Pre-operatively, 100% of the participants had painful sensation in the gums and post-operatively 100% of the patients observed a reduction in pain. Regarding the discomfort in eating food, before flap surgery, 90% of the participants had discomfort in eating food because of problems with the gums, whereas the number has been reduced to 46.6% post-surgery. With regard to psychological discomfort domain, 100% of the participants were conscious about the problems associated with gums pre-operatively whereas post-operatively 100% of the patients were satisfied with the gums. Before flap surgery, 99% of the participants were anxious about their gums. Post-operatively, anxiety levels were reduced to 100%. Overall in the psychological domain, the patients were completely satisfied (Table 2).

**Table 2:** Evaluation of patient-centered outcomes depicting functional limitations domain, physical pain domain, and psychological discomfort domain. Psychological discomfort domain showed statistically significant difference pre and post operatively(p<0.05). Taste perception showed a significant difference in functional limitations domain (p<0.05). Pain sensation showed significant difference in physical pain domain (p<0.05).

Domain	Questions	Pre-OP Flap Surgery					Post-OP Flap Surgery					p-value
		Strongly disagree	Disagree	Neutral	Agree	Strongly disagree	Strongly disagree	Disagree	Neutral	Agree	Strongly agree	
Functional Limitations	Have you had trouble pronouncing any words because of gum problem	-	-	6(20%)	5(16.7%)	19(63.3%)	-	1(3.1%)	18(60.3%)	11(36.6%)	-	>0.05
	Have you felt that your sense of taste has worsened because of gum problem	-	-	6(20%)	5(16.7%)	19(63.3%)	19(63.3%)	6(20%)	5(16.7%)	-	-	<0.05
Physical Pain	Have you had painful aching in your gums	-	-	1(3.1%)	18(60.3%)	11(36.6%)	1(3.1%)	18(60.3%)	11 (36.6%)	-	-	<0.05
	Have you found it uncomfortable to eat any foods because of problems with your gums	-	3(10%)	5(16.7%)	12(40%)	10(33.4%)	-	6(20%)	10(33.4%)	11(36.6%)	3(10%)	>0.05
Psychological discomfort	Have you been self-conscious because of your gums	-	-	2(6.6%)	11(36.6%)	17(56.8%)	18(60.3%)	11(36.6%)	1(3.1%)	-	-	<0.05
	Have you felt tense because of your gums	-	-	1(3.1%)	11 (36.6%)	18(60.3%)	17(56.8%)	11(36.6%)	2(6.6%)	-	-	<0.05

In the physical disability domain, there were two questions related to diet dissatisfaction and meal interruption. Ninety percent of the participants were unsatisfactory about their diet and interrupted meals and post-operatively, it was reduced only to 86.6%. This domain did not show a satisfactory result. In the psychological disability domain, there were questions related to inability to relax and embarrassment due to problems associated with gums. Pre-operatively, 100% of the participants were unable to relax due to problems associated with gums and after flap surgery, it was reduced to 63.4%. Ninety percent of the patients were feeling embarrassed before flap surgery and post-flap surgery, 100% of the participants were feeling satisfied and did not feel embarrassed. With regard to the social disability domain, 93.4% of the patients were feeling irritated due to problems in the gums before flap surgery, whereas after flap surgery only 6.6% of the patients were not irritated because of the problem with the gums. Before flap surgery, 100% of the participants found it difficult to do their usual jobs because of improper oral hygiene, whereas after flap surgery, 100% of the patients found it trouble-free to perform their regular jobs due to problems with the gums (Table 3).

**Table 3:** Evaluation of patient-centered outcomes depicting the physical disability domain, psychological disability domain, and social disability domain. The physical disability domain did not show a statistically significant difference pre and post operatively (p>0.05). The psychological disability domain showed a statistically significant difference pre and post operatively (p<0.05). The social disability domain showed a statistically significant difference in work restriction (p<0.05).

Domain	Questions	Pre-OP Flap Surgery					Post-OP Flap Surgery					p-value
		Strongly disagree	Disagree	Neutral	Agree	Strongly Agree	Strongly disagree	Disagree	Neutral	Agree	Strongly Agree	
Physical disability	Have you been your diet unsatisfactory because of problems with your gums	-	3(10%)	3(10%)	13(43.4%)	11(36.6%)	-	4(13.4%)	12(40%)	6(20%)	8(26.6%)	>0.05
	Have you had to interrupt meals because of problems with your gums	-	3(10%)	3(10%)	13(43.4%)	11(36.6%)	-	4(13.4%)	12(40%)	6(20%)	8(26.6%)	>0.05
Psychological disability	Have you found it difficult to relax because of problems with gums	-	-	2(6.6%)	17(56.8%)	11(36.6%)	2(6.6%)	17(56.8%)	11(36.6%)	-	-	<0.05
	Have you been a bit embarrassed because of gums	-	-	3(10%)	13(43.4%)	14(46.6%)	17(56.8%)	11(36.6%)	2(6.6%)	-	-	<0.05
Social disability	Have you been irritated because of gum problems	-	-	2(6.6%)	17(56.8%)	11(36.6%)	-	-	5(16.7%)	19(63.3%)	6(20%)	>0.05
	Have you felt difficult in doing usual jobs because of gum problems	-	-	3(10%)	13(43.3%)	14(46.6%)	17(56.8%)	11(36.6%)	2(6.6%)	-	-	<0.05

In the handicap domain, there were questions related to dissatisfaction with life and inability to function because of oral health problems. Pre-operatively, 93.4% of participants were unsatisfied with life because of problems with gums. Post-operatively, 16.7% gave a neutral response and the remaining 83.3% of participants were still unsatisfied about life because of problems with gums. With regard to the inability to function, 90% of the patients pre-operatively agreed that they were unable to function because of oral dysfunction, whereas post-operatively only 46.6% of patients agreed that they were able to function without any incongruity (Table 4).

**Table 4:** Patient-centered outcomes showing the handicap domain. Handicap domain did not  show a statistically significant difference pre and post operatively (p<0.05).

Domain	Questions	Pre-OP Flap Surgery					Post-OP Flap Surgery					p-value
		Strongly Disagree	Disagree	Neutral	Agree	Strongly Agree	Strongly Disagree	Disagree	Neutral	Agree	Strongly Agree	
Handicap	Have you felt that life in general was less satisfying because of problems with gums	-	-	2(6.6%)	17(56.8%)	11(36.6%)	-	-	5(16.7%)	19(63.3%)	6(20%)	>0.05
	Have you had to interrupt meals because of problems with your gums	-	-	3(10%)	13(43.4%)	14(46.6%)	-	-	14(46.6%)	13(43.4%)	3(10%)	>0.05

When patient-centered outcomes were compared pre- and post-surgically, there was statistical significance in terms of taste perception in the functional limitations domain, reduction in pain sensation in the physical pain domain, improvement in self-consciousness and reduction in anxiety levels in the psychological discomfort domain, diminution of feeling of embarrassment and enhancement inability to relax due to problem associated with gums in psychological disability domain, and improvement in workplace in the social disability domain (p<0.05).

## Discussion

The present study assessed the true end points and surrogate end points after surgical periodontal therapy which is paramount in elucidating the comprehensive impact of interventions on periodontal health and patient well-being. To our knowledge, this is the first study of its kind to utilize the OHIP-14 questionnaire in assessing patient-centered outcomes subsequent to periodontal flap surgery. 

When clinical parameters were compared pre- and post-surgery, there was a significant improvement in all the parameters, which is in accordance with previous studies [17-19]. Furthermore, it was also observed that there were improvement in taste perception, reduction in pain sensation, improvement in psychological comfort (self-consciousness and reduction in anxiety levels), improvement in psychological disability (diminution of the feeling of embarrassment and enhancement in ability to relax due to problem associated with gums) and enhancement in ability to relax because of the problem with gums post-surgery.

Lohiya et al. assessed the oral health-related quality of life (OHRQoL) after surgical periodontal therapy and observed that pain was markedly reduced after therapy and there was a positive correlation between surgical periodontal therapy and OHRQoL [20]. Needleman et al. studied a sample of patients with advanced periodontal disease who underwent surgical intervention and reported that there was significant reduction in pain and psychological discomfort post-operatively [21]. Similarly, Locker et al. conducted a longitudinal study among elderly patients aged from 75 to 83 years over a period of six months post-treatment and evaluated changes in OHRQoL using OHIP-14 and GOHAI (Geriatric Oral Health Assessment Index). Results demonstrated that both the questionnaires showed consistent improvement in various dimensions of OHRQoL with a significant reduction in pain, functional limitation, and psychological discomfort. These findings underscore the reliability and validity of OHIP-14 as a sensitive measure of treatment outcomes in periodontal therapy [22]. 

Shamim et al. evaluated the clinical parameters and OHRQoL using the OHIP-14 questionnaire among women with periodontal disease and also self-esteem was assessed using a Rosenberg Self-esteem Scale. The authors concluded that functional limitations and psychological disability had a significant impact on OHRQoL [23]. This infers that feeling of embarrassment and taste perception worsened with periodontal disease and were enhanced after periodontal therapy. Vivek et al. evaluated the impact of both non-surgical and surgical periodontal therapy on OHRQoL using the OHIP-14 questionnaire. The participants demonstrated 100% satisfaction in the workplace by boosting self-confidence. This infers that there was a significant impact on the social disability domain. The results showed that overall mean scores of OHRQoL improved after surgical periodontal therapy [24]. Similar results were demonstrated in various other studies [25-26]. Our results are in accordance with the previous studies.

However in the present study even after surgical periodontal therapy, patients were not happy with the pronunciation, diet restriction, and overall oral health. This study underscores the importance of an interdisciplinary approach in addressing the primary concerns of patients rather than solely focusing on treating the underlying cause. For instance, in cases of chronic periodontitis, it is essential to integrate prosthodontic and esthetic therapies which include full mouth rehabilitation and replacement of missing teeth along with periodontal treatment to address tooth loss, which may be the primary complaint of the patient. This holistic approach ensures the achievement of true endpoints, leading to substantial enhancement in pronunciation and diet restriction. This study also suggests the importance of identifying suitable true endpoint parameters for assessment following periodontal surgeries. In addition to evaluating surrogate parameters, it is essential to incorporate appropriate patient-centered true endpoints in any research endeavor to obtain a comprehensive and meaningful understanding of the efficacy of various periodontal techniques and therapies. 

The strength of the study includes utilizing a validated tool for assessing patient-based outcomes (OHIP-14), and longitudinal study design. However, there are a few limitations which include even though OHIP-14 provides a comprehensive assessment of OHRQoL, other psychometrically validated instruments may offer additional insights into specific aspects of patient well-being, warranting consideration in future research endeavors. The subjective nature of OHRQoL assessment using OHIP-14 may introduce bias, as responses are influenced by individual perceptions and experiences.

## Conclusions

In conclusion, surgical periodontal therapy plays a pivotal role in improving OHRQoL among patients with chronic periodontal disease. Utilizing OHIP-14 as an assessment tool enables a comprehensive evaluation of treatment outcomes, encompassing various dimensions of oral health impact. The positive correlations observed between surgical intervention and improvements in OHRQoL underscore the significance of addressing both clinical and patient-centered outcomes in periodontal care.
